# Mortality from cardiovascular disease in a cohort of Swedish seafarers

**DOI:** 10.1007/s00420-019-01486-5

**Published:** 2019-11-16

**Authors:** Helena P. Eriksson, Karl Forsell, Eva Andersson

**Affiliations:** 1 Occupational and Environmental Medicine, Sahlgrenska University Hospital, Gothenburg, Sweden; 2 Occupational and Environmental Medicine, Sahlgrenska Academy, University of Gothenburg, Gothenburg, Sweden; 3 Occupational and Environmental Medicine, Norrland University Hospital, Umeå, Sweden

**Keywords:** Seafarer, Mariner, Mortality, Coronary heart disease, Cerebrovascular disease

## Abstract

**Purpose:**

The aim of this study was to investigate whether Swedish seafarers have increased mortality from cardiovascular disease compared with the general population.

**Methods:**

Register-based longitudinal cohort study of 85,169 Swedish seafarers where all subjects with a minimum of 30 days service registered in the Seafarers’ Register 1985–2013 were included. Mortality from coronary heart disease, cerebrovascular disease and total mortality for comparison were analysed by calculating standardised mortality ratios (SMRs), with 95% confidence intervals (CIs). Mortality was further analysed by gender, duty on board, type of vessel, and over time.

**Results:**

There was no increase in either mortality from cardiovascular disease or total mortality for seafarers, who had worked solely on passenger ferries. Mortality from coronary heart disease and cerebrovascular disease was increased for male seafarers < 46 years old who had worked on different types of vessels, SMR 1.48 (95% CI 1.06–2.01) and SMR 1.93 (95% CI 1.16–3.02), respectively. Analysing the seafarers by duty showed significantly increased SMRs from coronary heart disease in males aged < 46 of the categories “deck crew” and “engine officer/crew (ever)”. The total mortality for seafarers who had worked on different types of vessels was increased; males SMR 1.05 (95% CI 1.02–1.09) and females SMR 1.17 (95% CI 1.04–1.30), but decreased over time.

**Conclusions:**

No increased mortality on passenger ferries but younger male seafarers on different types of vessels had increased mortality from cardiovascular disease. Reduction of hazardous occupational exposures onboard is important, such as shift work, stress and noise.

## Background

Previous studies have shown increased mortality and morbidity in seafarers compared with individuals in land-based occupations (Brandt et al. 1994, Rafnsson & Gunnarsdottir 1994; Jensen 1996; Mellbye & Carter 2017). There may be several reasons for this, such as lethal accidents (Oldenburg et al. 2016; Borch et al. 2012), cancer (Ugelvig Petersen et al. 2018), infectious diseases (Roberts & Carter 2016) and mental illness (Mellbye & Carter 2017). Cardiovascular disease (CVD) is another possible cause of both disease at sea (Alves et al. 2010) and sudden death among seafarers (Brandt et al. 1994; Oldenburg et al. 2016), and the chance of survival can be decreased due to long distance and prolonged time to advanced medical care (Jaremin & Kotulak 2003).

According to international studies, seafarers have increased risk factors for CVD, such as smoking, obesity and lack of physical exercise (Pougnet et al. 2013; Oldenburg 2014; Nittari et al. 2019). Other potential common risk factors at sea for CVD are shift work (Torquati et al. 2018), noise exposure (Skogstad et al. 2016; Forsell et al. 2017) and psychosocial stress (Theorell et al. 2016; Forsell et al. 2017). Moreover, Danish studies have found increased frequencies of hypertension and metabolic syndrome among seafarers (Tu & Jepsen 2016; Moller Pedersen & Jepsen 2013). The metabolic syndrome is likely linked, among other things, to lack of physical activity, as well as stress and shift work (Watanabe et al. 2018), and is an established risk factor for CVD (O’Neill & O’Driscoll 2015).

However, it is not known whether seafarers have an increased incidence of or mortality from CVD; there are very few studies performed and the results are conflicting (Hammar et al. 1992; Jaremin & Kotulak 2003; Roberts & Jaremin 2010): in a Swedish register-based study on different occupational groups, an increased incidence of myocardial infarction among deck officers was reported (Hammar et al. 1992). In a retrospective Polish study, the pre-hospital and 1-month mortality due to myocardial infarction was higher among seafarers compared with the general population (Jaremin & Kotulak 2003). A British study showed that mortality from CVD was generally lower among seafarers compared with the general population but an elevated risk of work-related CVD mortality was found among the crew members of North Sea offshore ships (Roberts & Jaremin 2010).

Thus, the seafarer’s occupation may involve exposure to several risk factors for CVD and likely varies among different occupational categories and among different types of vessels, but it is not clear whether this group have an increased risk of mortality from CVD; the results of the scarce previous studies are inconclusive. The aim of this study was to investigate whether Swedish seafarers have an increased mortality from CVD compared with the general population and to also analyse potential differences in mortality over time, between different types of work and different vessels.

## Methods

The study was a register-based longitudinal cohort study of Swedish seafarers. All Swedish merchant vessels with a gross tonnage of 20 or more are obliged to register the seafarers in their employ with the Seafarers’ Registry (SR) held by the Swedish Transport Agency. The start of the observation period corresponds to the start of the digitalization of the SR. In the register, sea services before 1985 have been entered retrospectively for seafarers who were active at this time. The subjects comprised all seafarers with a registered residency in Sweden who were registered with the SR as employed seafarers between 1 January 1985 and 31 December 2013, in total 102,870. Subjects with records of a minimum of 30 days of work on a vessel during that period were included in the cohort (*N* = 94,954). Those who lacked a personal identity number, i.e. foreign workers (*N* = 9587), were excluded as were subjects born before 1920 (*N* = 198) because they were at the usual age of retirement when the observation period of the study started. In total, there were 85,169 subjects after exclusions. Both men and women were included. The cohort will be used for other studies as well.

The study included record linkage between the national registers using the subjects’ personal identity number. The Swedish Transport Agency sent the SR data to Statistics Sweden for migration data and data on vital events. Statistics Sweden passed the data further to the National Board of Health and Welfare, who matched the cohort with the Swedish Cause of Death Register for the observation period 1985–2013 and anonymised the data before transferring them to us. Reference mortality data for the whole general population, stratified by gender, 5-year age groups, and 1-year calendar periods 1985–2013, were also retrieved from the National Board of Health and Welfare.

The information from the SR included dates of seafaring work, type of vessel and occupational position (duty) for each seafarer and for each period of service. On the grounds that seafarers often work on different types of vessels and in different types of positions during their working life, Table 1, the seafarers were categorised into different groups in our analysis. For type of vessel, we divided the subjects into two categories, seafarers who had worked *solely on passenger ferries* and seafarers who had worked *on different types of vessels,* as 53,.5% of the seafarers only had worked on passenger ferries and the remaining part on several types of vessels and work on passenger ferries is also likely more regular, shorter in duration and includes less chemical exposure. Based on the results presented in Table 1, duty on board was divided into four categories: *solely service* means only having served in the service section of a vessel; *deck officer (ever)* means ever having worked as a deck officer; *engine officer/crew (ever)* means ever having worked in the engine room as officer or crew; and *deck crew* means having worked as deck crew and possibly also having served in the service section. The seafarers were subdivided into groups based on *time registered as a seafarer*, < 1 year, 1–5 years, 5–10 years, 10–20 years, and > 20 years. Numbers of individuals in each subgroup are presented in Table 2.

**Table 1:** The cohort of Swedish seafarers with sea service any time during 1985–2013, showing how common it was to have worked on different types of vessels and with different duties on board

If ever type what other types also, % of *N*	Only one type%	Sea service on different types of vessels^a^					*N*
		Type A%	Type F%	Type O%	Type P%	Type T%	
Ever type A	10.0		3.7	47.5	68.1	82.3	21,620
Ever type F	0.8	78.7		78.8	77.8	95.8	1023
Ever type O	10.5	58.4	4.6		70.9	76.8	17,581
Ever type P	65.3	21.1	1.1	17.9		30.0	69,848
Ever type T	16.6	55.5	3.1	42.2	65.5		32,047

If ever duty what other duties also, % of N	Only one duty%	Sea service with different duties					N
		Deck officer%	Deck crew%	Engine officer%	Engine crew%	Service%	
Ever deck officer	14.9		79.6	5.9	8.0	18.9	10,212
Ever deck crew	48.5	26.2		5.9	13.8	20.9	31,003
Ever engine officer	15.1	9.2	27.8		76.4	17.2	6561
Ever engine crew	30.9	6.6	34.5	40.4		18.7	12,398
Service	84.2	3.8	12.6	2.2	4.5		51,203

^a^Type A—different kinds of tankers, type F—fishing vessels, type P—passenger ferries, type T—transportation vessels, type O—working vessels and other vessels

**Table 2:** The cohort of Swedish seafarers from 1985 to 2013, in numbers and person-years, age at start of sea service, years of seafarer service, numbers of years in different categories of seafarer work, and starting work as a seafarer in 1985 or later, as well as gender, type of vessel, and duty

	Sea service on different types of vessels					Sea service solely on passenger ferries					All
	Deck officer (ever)	Deck crew	Engine officer/ crew (ever)	Solely service	Total	Deck officer (ever)	Deck crew	Engine officer/ crew (ever)	Solely service	Total	
Male seafarers, N	9039	10,661	10,788	3149	33,637	718	4562	1637	14,059	20,976	54,613
*Person*-*years*	*195,369*	*193,361*	*214,489*	*58,995*	*662,213*	*10,730*	*60,871*	*25,986*	*215,306*	*312,894*	*975,107*
Sea start age, yrs, median	19	19	19	22	19	44	23	26	25	25	22
Q1–Q3	17–25	17–26	17–24	18–28	17–25	33–54	19–36	20–40	21–31	21–33	18–29
Seafarer yrs, median	26	5.3	15	8.9	14	7.2	1.8	3.3	2.0	2.1	6.2
Q1–Q3	14–40	1.5–17	4.0–32	1.1–25	3.4–30	2.8–13	0.6–4.7	0.9–11	0.6–5.5	0.6–5.9	1.4–22
< 1 yr seafarer, N	189	2093	1287	724	4293	88	1644	454	4921	7107	11,400
1–5 yrs seafarer, N	459	3088	1740	601	5888	181	1853	519	5377	7930	13,818
5–10 yrs seafarer, N	884	1575	1313	316	4088	183	540	237	1745	2705	6793
10–20 yrs seafarer, N	1871	1639	1901	476	5887	187	332	215	1222	1956	7843
> 20 yrs seafarer, N	5636	2266	4547	1032	13,481	79	193	212	794	1278	14,759
Males starting in/after 1985, N	3317	6770	5155	1634	16,876	671	4252	1398	12,893	19,214	36,090
*Person*-*years*	*53,913*	*96,165*	*74,543*	*22,947*	*247,568*	*9543*	*52,692*	*19,704*	*185,932*	*267,871*	*515,439*
Female seafarers, N	412	1461	282	3775	5930	43	2359	88	22,136	24,626	30,556
*Person*-*years*	*7975*	*19,331*	*3967*	*76,416*	*107,690*	*596*	*25,827*	*1286*	*363,114*	*390,824*	*498,514*
Sea start age, yrs, median	21	19	19	22	21	26	22	22	23	23	23
Q1–Q3	19–24	17–23	18–21	19–29	18–27	20–41	19–28	19–29	21–28	20–28	20–28
Seafarer yrs, median	11	2.8	5.1	5.6	5.0	9.2	2.2	4.0	2.0	2.0	2.3
Q1–Q3	7.4–17	1.4–7.0	1.7–9.6	1.3–15	1.5–13	4.8–13	0.7–4.5	1.2–9.6	0.5–5.2	0.6–5.2	0.6–6.4
< 1 yr seafarer, N	7	252	51	776	1086	2	739	19	7588	8348	9434
1–5 yrs seafarer, N	51	698	89	1024	1862	9	1114	28	8785	9936	11,798
5–10 yrs seafarer, N	128	269	76	627	1100	12	350	23	2892	3277	4377
10–20 yrs seafarer, N	160	160	57	706	1083	17	135	14	1778	1944	3027
> 20 yrs seafarer, N	66	82	9	642	799	3	21	4	1093	1121	1920
Females starting in/after 1985, N	218	1212	234	2172	3836	43	2326	81	19,998	22,448	26,284
*Person*-*years*	*2893*	*12,782*	*2653*	*35,545*	*53,873*	*596*	*24,903*	*1083*	*30,6947*	*333,530*	*38,7403*

*N*  number, *Q1*  first quartile, *Q3*  third quartile

Outcomes studied were cardiovascular diagnoses from the Swedish Cause of Death Register and total mortality from Statistics Sweden. Outcomes were classified according to the diagnostic codes of the International Statistical Classification of Diseases and Related Health Problems, 8th–10th revisions (ICD-8 to ICD-10). Thus, coronary heart disease (CHD) was defined as ICD-8/9 codes 410–414 and as ICD-10 codes I20–I25, myocardial infarction as ICD-8/9 410 and ICD-10 I21. Cerebrovascular disease, including both ischaemic stroke and intracerebral bleeding, was defined as ICD-8/9 430–438 and ICD-10 I60–I69. Ischaemic stroke was defined as ICD-8/9 433–434 and ICD-10 I63–I64 and intracerebral bleeding as ICD-8/9 431 and ICD-10 I61. Total mortality was studied to compare it with mortality from CVD.

### Statistical methods

Age at first sea service, and seafarer years are presented as medians with first and third quartiles (Q1, Q3) (Table 2). The person-years at risk were calculated starting from first time of work at sea in the SR until first emigration, time of death or the end of follow-up, 31 December 2013. The person-years were stratified by gender, 5-year age groups, and 1-year calendar periods. The expected number of deaths for these strata was calculated using the general Swedish population as a reference. Standardised mortality ratios (SMRs) were calculated and 95% confidence intervals (CIs) determined with the assumption of a Poisson distribution of the observed deaths. The SMR was calculated stratified for gender, type of vessel, position held, and time registered as a seafarer. The SMR was also calculated for person-years in different age groups: < 46, 46–55, 56–65, and > 65 years. Further analyses were done on seafarers who started their sea service before 1985 and after 1985, respectively, (when the digitalization was done and the follow-up period started) and by dividing the observation period into two periods, 1985–1999 and 2000–2013. The analyses were performed using STATA SE release 14 (Stata Statistical Software, College Station, TX, USA) and SAS 9.4 (SAS Institute, Cary, NC, USA).

The study was approved by the Regional Ethical Board of Gothenburg (193-13).

## Results

### Seafarers having worked solely on passenger ferries

In seafarers who had only worked on passenger ferries, there was no increased total mortality or increased mortality from CHD or cerebrovascular disease, compared with the general population, for either males or females (Table 3). For solely service personnel on passenger ferries, the main type of duty on these vessels, SMR for CHD was 0.75 (95% CI 0.56–0.99) for males and 0.50 (95% CI 0.33–0.71) for females (data not shown).

**Table 3:** Mortality from cardiovascular disease and total mortality, 1985–2013, among Swedish seafarers working solely on passenger ferries vs serving on different types of vessels, compared with the general population

	O	Age < 46 yrs		O	Age 46–55 yrs		O	Age 56–65 yrs		O	Age > 65 yrs		O	Total	
		SMR	95% CI		SMR	95% CI		SMR	95% CI		SMR	95% CI		SMR	95% CI
*Male seafarers solely on passenger ferries*															
Total mortality	219	0.98	0.86–1.12	157	1.03	0.88–1.21	138	0.70	0.59–0.83	189	0.58	0.50–0.67	703	0.78	0.73–0.84
Coronary heart disease	6	0.50	0.18–1.09	26	1.01	0.66–1.48	24	0.56	0.36–0.83	37	0.53	0.37–0.73	93	0.62	0.50–0.76
Myocardial infarction	2		(7.5)	12	0.74	0.38–1.29	17	0.63	0.37–1.01	19	0.48	0.29–0.75	50	0.55	0.41–0.73
Cerebrovascular disease	3	0.65	0.13–1.89	7	1.12	0.45–2.31	6	0.64	0.24–1.40	15	0.61	0.34–1.01	31	0.69	0.47–0.98
*Female seafarers solely on passenger ferries*															
Total mortality	127	0.84	0.70–0.99	93	0.89	0.72–1.09	89	0.77	0.62–0.94	123	0.46	0.38–0.55	432	0.68	0.61–0.74
Coronary heart disease	1		(3.6)	2		(6.2)	11	0.95	0.47–1.69	16	0.39	0.22–0.63	30	0.48	0.32–0.68
Myocardial infarction	0		(2.4)	1		(4.1)	7	0.92	0.37–1.90	8	0.35	0.15–0.68	16	0.43	0.25–0.70
Cerebrovascular disease	1		(4.6)	5	1.07	0.35–2.49	4	0.70	0.19–1.80	12	0.48	0.25–0.84	22	0.55	0.35–0.84
*Male seafarers on different vessels*															
Total mortality	557	**1.36**	**1.25–1.48**	618	**1.21**	**1.12–1.31**	1116	**1.19**	**1.12–1.26**	1656	0.88	0.84–0.92	3947	**1.05**	**1.02–1.09**
Coronary heart disease	41	**1.48**	**1.06–2.01**	100	1.00	0.81–1.21	232	1.00	0.88–1.14	356	0.84	0.76–0.93	729	0.93	0.87–1.00
Myocardial infarction	23	1.29	0.82–1.94	55	0.85	0.64–1.10	130	0.88	0.74–1.04	191	0.78	0.67–0.89	399	0.84	0.76–0.92
Cerebrovascular disease	19	**1.93**	**1.16–3.02**	17	0.78	0.46–1.25	50	1.05	0.78–1.38	110	0.75	0.62–0.90	196	0.87	0.75–1.00
Stroke	0		(1.5)	3	0.61	0.13–1.79	12	0.74	0.38–1.30	56	0.82	0.62–1.06	71	0.78	0.61–0.98
Cerebral haemorrhage	9	**2.18**	**1.00–4.13**	8	0.81	0.35–1.59	25	1.37	0.89–2.03	23	0.73	0.46–1.10	65	1.02	0.79–1.30
*Female seafarers on different vessels*															
Total mortality	47	1.20	0.88–1.60	60	**1.40**	**1.07–1.81**	64	1.12	0.87–1.44	163	1.10	0.94–1.29	334	**1.17**	**1.04–1.30**
Coronary heart disease	0		(1.0)	3	1.13	0.23–3.29	9	1.43	0.65–2.72	29	1.23	0.82–1.76	41	1.22	0.87–1.65
Myocardial infarction	0		(0.7)	2		(1.8)	3	0.72	0.15–2.10	13	0.94	0.50–1.60	18	0.88	0.52–1.38
Cerebrovascular disease	1		(1.3)	4	2.02	0.55–5.17	2		(2.9)	16	1.15	0.65–1.86	23	1.14	0.72–1.71

Standardised mortality ratios (SMRs) are given with observed cases (O) and 95% confidence intervals (CIs), by gender and age at death. Where fewer than three cases were reported, expected values are shown in parentheses

Significantly elevated SMRs are marked in bold

### Seafarers having worked on different types of vessels

When analysing all of the seafarers who had worked on different types of vessels as one group, there were no significantly increased SMRs for CHD or cerebrovascular disease for males or females (Table 3). The SMR for total mortality was, however, slightly increased for both males and females (Table 3).

Analysing male seafarers by age showed that, in the age group < 46 years, the SMR for CHD was significantly increased, 1.48 (95% CI 1.06–2.01) (Table 3), as was the SMR for total mortality, 1.36 (95% CI 1.25–1.48) (Table 3). The results also differed according to number of years registered with the SR: for male seafarers who were < 46 years old and had been registered for 10–20 years, the SMR for CHD was 2.46 (95% CI 1.31–4.20) (Table 4). Moreover, when evaluating mortality according to the seafarers’ duty on board, it was the categories male deck crew and male engine officer/crew (ever) < 46 years that had significantly increased SMRs for CHD (Table 4). When we further analysed the seafarers according to start of sea service, we observed that, for male seafarers < 46 years who started before 1985, the SMR for CHD remained significantly increased (Table 4); for seafarers who started their sea service after 1985, the SMR for CHD was similar, compared to the subjects who started before 1985 but no longer significantly increased (Table 4).

**Table 4:** Mortality from cardiovascular disease, 1985–2013, among male Swedish seafarers serving on different types of vessels (*N* = 33,637) compared with the general population

Male seafarers on different vessels	O	Age < 46 yrs		O	Age 46–55 yrs		O	Age 56–65 yrs		O	Age > 65 yrs		O	Total	
		SMR	95% CI		SMR	95% CI		SMR	95% CI		SMR	95% CI		SMR	95% CI
Coronary heart disease															
Deck officer, ever	6	0.72	0.27–158	32	0.92	0.63–1.30	73	0.80	0.62–1.00	144	0.73	0.61–0.85	255	0.77	0.67–0.87
Deck crew (not officer)	17	**2.21**	**1.29–3.54**	23	1.06	0.67–1.60	51	1.26	0.94–1.65	50	0.85	0.63–1.12	141	1.09	0.92–1.29
Engine officer/crew, ever	17	**1.86**	**1.08–2.98**	34	1.00	0.69–1.39	80	1.03	0.82–1.28	132	1.03	0.86–1.22	263	1.06	0.93–1.19
Solely service	1		(2.6)	11	1.13	0.56–2.01	28	1.28	0.85–1.85	30	0.79	0.54–1.13	70	0.97	0.76–1.23
< 1 yr seafarer	3	1.37	0.28–4.01	3	0.74	0.15–2.16	1		(6.7)	5	0.67	0.22–1.56	12	0.59	0.30–1.03
1–5 yrs seafarer	5	2.03	0.66–4.74	2		(4.6)	6	0.81	0.30–1.76	6	0.59	0.22–1.29	19	0.77	0.47–1.21
5–10 yrs seafarer	3	1.35	0.28–3.93	3	0.64	0.13–1.86	7	0.90	0.36–1.86	7	0.53	0.21–1.09	20	0.72	0.44–1.10
10–20 yrs seafarer	13^a^	**2.46**	**1.31–4.20**	18	1.27	0.75–2.01	31^b^	1.42	0.96–2.01	22	0.69	0.43–1.05	84	1.15	0.92–1.42
> 20 yrs seafarer	17	1.09	0.64–1.75	74	1.02	0.80–1.28	187	1.00	0.86–1.15	316	0.88	0.78–0.98	594	0.93	0.86–1.01
Cardiovascular disease															
Start before 1985															
Coronary heart disease	31	**1.49**	**1.01–2.11**	94	1.08	0.87–1.32	215	1.03	0.89–1.17	341	0.85	0.76–0.95	681	0.95	0.88–1.02
Myocardial infarction	17	1.26	0.73–2.02	52	0.92	0.68–1.20	120	0.89	0.74–1.07	182	0.78	0.67–0.90	371	0.85	0.76–0.94
Start in/after 1985															
Coronary heart disease	10	1.45	0.70–2.67	6	0.46	0.17–1.00	17	0.77	0.45–1.23	15	0.65	0.36–1.08	48	0.74	0.54–0.98
Myocardial infarction	6	1.40	0.51–3.04	3	0.37	0.08–1.07	10	0.74	0.35–1.35	9	0.68	0.31–1.29	28	0.71	0.47–1.03
Death in 1985–1999															
Coronary heart disease	30	**1.60**	**1.08–2.28**	59	0.95	0.72–1.22	100	0.84	0.68–1.02	90	0.90	0.72–1.10	279	0.93	0.82–1.04
Myocardial infarction	17	1.40	0.81–2.24	36	0.86	0.60–1.19	60	0.74	0.57–0.95	54	0.81	0.61–1.05	167	0.83	0.71–0.96
Cerebrovascular disease	16	**2.43**	**1.39–3.94**	10	0.83	0.40–1.52	24	1.09	0.70–1.62	14	0.54	0.30–0.91	64	0.96	0.74–1.23
Death in 2000–2013															
Coronary heart disease	11	1.23	0.61–2.20	41	1.08	0.78–1.47	132	1.18	0.99–1.40	266	0.82	0.73–0.93	450	0.94	0.85–1.03
Myocardial infarction	6	1.07	0.39–2.33	19	0.83	0.50–1.29	70	1.05	0.82–1.32	137	0.77	0.64–0.90	232	0.85	0.74–0.96
Cerebrovascular disease	3	0.93	0.19–2.71	7	0.73	0.29–1.50	26	1.01	0.66–1.49	96	0.79	0.64–0.97	132	0.83	0.69–0.98

Standardised mortality ratios (SMRs) are given with observed cases (O) and 95% confidence intervals (CIs), by age at death. For coronary heart disease according to duty, and number of years as a seafarer; for cardiovascular diseases according to start of sea service and time period of death. Where fewer than three cases were reported, expected values are shown in parentheses

Significantly elevated SMRs are marked in bold

Myocardial infarction: ^a^*N* = 9; SMR 2.66; 95% CI 1.22–5.05; ^b^*N* = 23; SMR 1.68; 95% CI 1.07–2.52

Finally, dividing the observation period into two periods showed that total mortality for males and females and mortality from CVD for males < 46 years remained significantly increased for the years 1985–1999. For the observation period 2000–2013, the SMRs for CVD decreased and became insignificant, even though there was a borderline increased mortality from CHD for men aged 56–65 years (Table 4). The total mortality for the seafarers having served on different types of vessels as one group decreased; however, it was significantly increased for male seafarers in the age groups 46–55 and 56–65 years (SMR 1.27 (95% CI 1.13–1.42) and SMR 1.30 (95% CI 1.21–1.40), respectively) (not shown in the tables).

In addition, male seafarers < 46 years had a significantly increased mortality from cerebrovascular disease, SMR 1.93 (95% CI 1.16–3.02). The mortality due to cerebral haemorrhage was also increased in this group, SMR 2.18 (95% CI 1.00–4.13) (Table 3). When analysing according to years of register in the SR and type of duty, the mortality from cerebrovascular disease was insignificantly increased among them registered for 10–20 years, and mainly the duty deck crew (data not shown).

For women, the SMR for mortality due to CHD was 1.22 (95% CI 0.87–1.65) with the highest but insignificant SMRs for the ages 56–65 years and > 65 years, with work as a seafarer 10–20 years and > 20 years among all duties except engine room were very few had worked (Table 3 and data not shown). SMR due to cerebrovascular disease for women was 1.14 (95% CI 0.72–1.71), mostly occurring in the age > 65 years and in the category solely service who had served > 10 years at sea (Table 3 and data not shown). The total mortality was increased for the ages 46–55, SMR 1.40 (95% CI 1.07–1.81) (Table 3).

## Discussion

Our study shows that there was an increased mortality from CHD and cerebrovascular disease in relatively younger male seafarers having worked on different types of vessels, especially those with several years of service. We could not clearly show any significantly increased mortality from CHD or cerebrovascular disease among women. Our results demonstrate that Swedish seafarers on different types of vessels, both men and women, had a significantly but modestly increased total mortality. Our results corroborate earlier international studies regarding increased general mortality among seafarers (Brandt et al. 1994; Rafnsson & Gunnarsdottir 1994; Jensen 1996).

We had no information regarding location of death. It is possible that the increased mortality from CVD could partially be attributed to long distance to qualified health care if the illness occurs at sea. In a Polish study, pre-hospital mortality from myocardial infarction was higher, but the incidence was not increased, in seafarers compared with the general working population (Jaremin & Kotulak 2003). The authors concluded that work-related factors reduce survival at sea in the case of a myocardial infarction. However, the long distance is not likely to explain the entire increase as in our study the high mortality figures were also related to seafarer age, type of vessel, and length of registration as a seafarer.

The mortality from CHD was not significantly increased for seafarers who started their sea service after 1985, or for the observation period 2000–2013, but this may partly have been due to lack of power. The modest increase in total mortality decreased over time. There could be several reasons for that; hopefully, the work environment has improved, smoking has probably decreased especially among men, and the length of time as seafarer has overall decreased. In a Danish study analysing mortality in seafarers 1986–2009, the total mortality diminished over time; however, there was no clear decrease in mortality from heart diseases (Borch et al. 2012).

Seafarers often serve on several types of vessels during their professional career, and the occupational exposure is likely different on different types of vessels. Taking this into account, although it was not possible in this study to separately analyse mortality for each type of vessel, we divided the seafarers into two groups; those who had worked on different types of vessels and those who had worked solely on passenger ferries. The seafarers who had worked on passenger ferries only, had no increased mortality, but they had worked shorter time as seafarers and our study population had few seafarers who had both served before 1985 and worked solely on passenger ships, which may have affected the internal validity of this result. It should, however, be noted that the exposure patterns of passenger ferries and other types of vessels differ substantially, including more regular work, and less chemical exposure on passenger ferries. A Norwegian register study detected an increased overall mortality, but no significantly increased mortality from CVD, for seafarers working on tankers compared with seafarers working on other ships. The increased risk was related to work as a mate; there was no increased risk for those working as captains (Moen et al. 1994).

In our study, mortality from CHD was increased among subjects who had worked 10–20 years at sea. After 20 years of registered seamanship, the mortality from CHD declined. This could have been due to a healthy worker effect and to the earlier increased mortality in the cohort causing diminished cohort mortality later in life. The earlier increase in mortality is likely also to have been caused by competing risks such as lethal accidents and cancer diseases, in addition to CVD. Competing risks and earlier increased mortality can also be applied to male seafarers > 65 years of age who had no increased mortality. In an Icelandic study analysing mortality among seafarers, the mortality from ischaemic heart disease was the highest among seafarers who had worked for 8–10 years, SMR 1.56 (95% CI 1.14–2.7); but thereafter the mortality decreased (Rafnsson & Gunnarsdottir 1994).

In our study it was the categories younger male deck crew and male engine officer/crew (ever) that had significantly increased mortality from CHD. In a Danish retrospective cohort study on seafarers from 1970 to 1985, a significantly increased mortality rate from CHD was reported only for the engine crew, compared with the general population (Brandt et al. 1994). The occupational exposure is probably different among different positions at sea due to different work tasks in different locations with varying occupational hazards.

For the women in our study, there was an insignificant tendency towards increased mortality from CHD at a later age compared with the men. Women generally develop CHD later in life compared with men (Bello & Mosca 2004). The insignificant result for the females in our study may be due to lack of statistical power; there are fewer female seafarers and hence, there were fewer females in the cohort.

This study has several strengths, such as high validity, it being a large longitudinal cohort study with a long follow-up time and including information on type of work and type of vessel for each service for each individual. Also, we had access to data on both total mortality and mortality from CVD from the Swedish national registers which have high coverage and good quality.

Our study has several limitations. It was not possible to obtain individual smoking data or other individual medical data regarding risk factors for CVD since this was a register study. International studies have shown increased risk of obesity and the metabolic syndrome among seafarers (Moller Pedersen & Jepsen 2013; Nittari et al. 2019), but we do not know if this could be applied to Swedish seafarers. In 1977 in Sweden, 67% of male deck officers, 51% of male engine officers and 35% of male engine crew were smokers, compared with 44% of the general male population (Nilsson 1998). The increased risk for CHD associated with cigarette smoking, however, decreases relatively fast after smoking cessation (Teo et al. 2006). In a Swedish questionnaire survey from 2014 answered by 1972 seafarers, 11% of the respondents were current daily smokers, with no marked difference between men and women. Among the service personnel, the proportion of smokers was higher, 23%. In the Swedish general population, 9% of men and 11% of women were daily smokers in 2015, according to the Public Health Agency of Sweden. One of our inclusion criteria, a minimum of 30 days of work on a vessel, could be considered short and a limitation of the study.

Our results show an increased mortality from CVD at a relatively young age for male seafarers who had served on different types of vessels, particularly those who had served as seafarers for several years. Our results also show a disparity in mortality between subjects working on passenger ferries and subjects working on other types of vessels, which indicates a difference in the work environment and calls for improvements such as optimization of the shift work schedules and reducing noise, stress and chemical exposure.

The modestly increased mortality among Swedish seafarers seems to have decreased over time, but further studies are needed on the specific causes of the increased total mortality among seafarers.
